# New Insights into High-Fat Diet with Chronic Diseases

**DOI:** 10.3390/nu15184031

**Published:** 2023-09-18

**Authors:** Xiaoyu Wang, Rui Song, Maëlys Clinchamps, Frédéric Dutheil

**Affiliations:** 1 College of Food Science & Nutritional Engineering, China Agricultural University, Beijing 100083, China; songrui_123@cau.edu.cn; 2 Key Laboratory of Precision Nutrition and Food Quality, Department of Nutrition and Health, China Agricultural University, Beijing 100193, China; 3 CNRS, LaPSCo, Physiological and Psychosocial Stress, University Clermont Auvergne, F-63000 Clermont-Ferrand, France; maelysclinchamps@gmail.com (M.C.); fred_dutheil@yahoo.fr (F.D.)

## 1. Introduction

Chronic diseases, encompassing conditions such as heart disease, cancer, and diabetes, represent a significant global health challenge and are the leading causes of mortality worldwide. Extensive research has been dedicated to investigating the relationship between dietary fat consumption and the risk of developing chronic diseases over the years. It is well-established that lifestyle modifications, particularly dietary choices, hold substantial potential for preventing and mitigating the risk of these chronic conditions. This Special Issue of *Nutrients*, entitled “High Fat Diet with Chronic Diseases”, aims to contribute to our comprehension of the potential underlying mechanisms connecting high-fat diets to chronic diseases. Furthermore, this Special Issue places a strong emphasis on the development of effective therapeutic strategies for managing and combating these conditions. The studies in this issue are grouped into four categories: (i) obesity and metabolic disorders, (ii) pancreatic health and diabetes, (iii) skeletal and muscle health, and (iv) neurological and cognitive function. While these topics seem distinct, they share an underlying theme: the complex interplay between dietary factors, obesity, and their consequences on health.

## 2. Obesity and Metabolic Disorders

Several studies collectively provide a comprehensive exploration into the area of obesity and metabolism-related diseases. While each study explores distinct subjects, their collective findings reveal intricate underlying connections, with a notable emphasis on dietary supplements. The study authored by S. Heo et al. delves into the potential of *Cassia mimosoides* var. *nomame* Makino extract (EECM) as a means to combat obesity [1]. EECM demonstrates its efficacy by inhibiting adipogenesis and lipogenesis, primarily through the AMP-activated protein kinase pathway. C. Chou et al. scrutinize the influence of high fructose intake on lipolysis and reveal how valsartan and amlodipine can effectively mitigate lipolysis through PU.1 inhibition, underscoring the potential of these drugs in addressing fructose-induced obesity [2]. Y. Wang et al. explore the effects of coarse cereals on lipid metabolism and oxido-inflammatory responses [3]. Oats and tartary buckwheat, rich in polyphenols and dietary fiber, exhibit their potential in modulating gut microbiota, improving lipid metabolism, and reducing oxidative stress and inflammation in rats fed a high-fat diet. Additionally, the significance of probiotics in alleviating obesity is pivotal. The study by C. Wang et al. investigates the effects of fermented milk containing *Bifidobacterium animalis* subsp. *lactis* MN-Gup (MN-Gup), which could alleviate HFD-induced body weight gain, epididymal fat deposition, adipocyte hypertrophy, dyslipidemia and inflammation, which may play important roles in the mechanism underlying the alleviation of obesity [4]. Meanwhile, Y. Huo et al. found that *Bifidobacterium animalis* subsp. *lactis* A6 enhances adipose tissue fatty acid β-oxidation (FAO) to mitigate obesity development by increasing acetate levels and activating the GPR43-PPARα signaling pathway in mice [5].

In addition, two articles specifically delve into lipid metabolism in unique populations. The study by R. Lugarà et al. investigates the impact of a Western diet on gestating and lactating sows, revealing signs of lean metabolic syndrome characterized by disrupted cholesterol levels, decreased IGF1 levels, and indications of hepatic damage [6]. Spirulina supplementation in this context partially mitigates these effects, hinting at its potential to counteract some of the negative outcomes associated with a Western diet. R. Song et al. review lipid metabolism in the elderly, emphasizing how age-related changes in digestion, absorption, and lipid utilization contribute to excess fat accumulation [7]. This accumulation is linked to chronic lipid-related diseases in older individuals, and understanding altered lipid metabolism in these populations is crucial for developing targeted interventions to address age-related chronic diseases. In summary, these studies collectively contribute to our understanding of obesity and metabolic disorders by examining both dietary supplements and their potential intervention on fat metabolism. They provide valuable insights into potential therapeutic strategies and the intricate mechanisms underlying these conditions, spanning various populations.

## 3. Pancreatic Health and Diabetes

Three papers shed light on glucose homeostasis and diabetes. M.K. Brahma et al. study the impact of Nova1 or Bim deficiency in pancreatic β-cells on diabetes and obesity in mice [8]. The deletion of Nova1 or Bim did not affect glucose homeostasis or diabetes development in response to multiple low-dose streptozotocin (MLD-STZ)-induced β-cell dysfunction or high-fat diet-induced insulin resistance. Another study explored the effects of a long-term high-fat, high-fructose (HFHF) diet on diabetes development in rats. Rats on HFHF diets exhibited metabolic disorders, changes in pancreatic islet size, and increased insulin levels. Authors Y. Zhao et al. suggest that long-term HFHF diets and age-related structural and transcriptomic changes in pancreatic islets may contribute to type 2 diabetes development [9]. The review authored by Y. Qi et al. focuses on the role of gut microbiota in high-fat diet-induced diabetes [10]. It highlights the importance of diet and gut microbiome in the development of diabetes and suggests that modifying the gut microbial community through probiotic and prebiotic approaches could be a promising strategy for diabetes prevention. Together, these papers provide valuable insights into the complex relationship between glucose homeostasis, diet, and diabetes, offering potential directions for future research and therapeutic interventions.

## 4. Skeletal and Muscle Health

Two articles are related to diet-induced obesity and skeletal/muscle health. The study by E. Nebot et al. investigates how obesity affects bone health [11]. It establishes that obesity might lead to increased bone mineral density, yet also pose a significant risk for fractures. Interventions like caloric restriction and exercise effectively counter bone structure and mineral density changes and improve body composition by reducing body fat and increasing lean body mass. Y. Zou et al. employed a zebrafish model to explore the impact of a high-fat diet on muscle mitochondrial function [12]. Obesity was linked to reduced exercise capacity, decreased skeletal muscle fiber cross-sectional area, and elevated expression of atrophy-related markers. The study demonstrated that mitochondrial dysfunction contributes to muscle atrophy in obesity. These findings collectively emphasize the intricate connection between dietary factors, obesity, and their consequences on bone and muscle health.

## 5. Neurological and Cognitive Function

Two studies explore the connection between obesity and cognitive function. Y. Liu et al. found that high-calorie food-cues impaired food-related conflict control [13]. Participants displayed slower reaction times and reduced accuracy when dealing with high-calorie food images. EEG data exhibited a notable reduction in N2 amplitudes and a decline in theta power when exposed to high-calorie foods, which serves as an indicator of cognitive impairments. The study by H. Zhang et al. investigated cognitive decline in obese mice, highlighting the beneficial effects of swimming [14]. Obesity led to cognitive impairment, but an 8-week swimming regimen mitigated this decline by reducing inflammation, inhibiting the JNK/IRS-1/PI3K/Akt pathway, and activating the PGC-1α/BDNF pathway. Overall, these studies underscore the detrimental impact of high-calorie food cues on cognitive function and emphasize the potential cognitive benefits of exercise interventions in combating obesity-related cognitive decline.

## 6. Conclusions

This Special Issue of *Nutrients* delves into the intricate relationship between dietary fat intake and chronic diseases. Understanding these relationships is crucial for developing effective strategies to combat diet-induced chronic diseases and improve overall health. These papers emphasize the role of nutritional supplements in addressing obesity and metabolic disorders while highlighting the significance of tailored interventions in unique populations’ lipid metabolism research. They include findings on glucose regulation and diabetes, encompassing studies on pancreatic β-cell and gut microbiota in diabetes development, and opening up promising pathways for future research and therapeutic interventions. Studies on obesity’s effects on bone, muscle health, and cognitive function offer insights into interventions like exercise and caloric restriction. In summary, this Special Issue serves as a platform to enhance our understanding of the intricate interplay between dietary fat intake and chronic diseases, offering insights into potential avenues for prevention and treatment. 
